# A Case Report of Acute Saddle Pulmonary Embolism in Prader-Willi Syndrome

**DOI:** 10.7759/cureus.57466

**Published:** 2024-04-02

**Authors:** Austin Rahman, Amar Mittapalli, Marlee Goldstein

**Affiliations:** 1 Emergency Medicine, Lake Erie College of Osteopathic Medicine, Bradenton, USA; 2 Emergency Medicine, AdventHealth, Tavares, USA

**Keywords:** deep vein thrombosis (dvt), saddle pulmonary embolism, comorbid obesity, xray, ekos catheter, tachypnea, prader-willi syndrome, acute pulmonary embolism, pulmonary embolism

## Abstract

Prader-Willi syndrome (PWS) is an exceedingly rare congenital syndrome of chromosome 15 that presents multiple comorbidities in said individuals. The associated quality of life for those with the disease is often severely diminished; more tragically, mortality associated with the disease is also increased. Pulmonary embolism (PE) is highly associated with mortality and has been shown to be more prevalent in patients with PWS. This case report details a patient with PWS who survived an acute saddle PE and looks to bring more clinical knowledge that can be applied when dealing with individuals with PWS.

## Introduction

Prader-Willi syndrome (PWS) is a sporadic genetic syndrome due to methylation at 15q11.2-q13 [1,2]. This genetic mutation has multiple ramifications for the human system, but it also has classical characteristics such as hypogonadism, morbid obesity, hyperphagia, and restrictions in growth and cognition [1,2]. These symptoms, especially morbid obesity, cause drastic changes in the quality of life of individuals with PWS; the average life expectancy of a PWS patient is roughly 30 years [3]. Furthermore, most deaths associated with PWS are due to respiratory failure [3]. However, pulmonary embolism (PE) makes up a significant portion of the deaths related to PWS [3]. PE is a life-threatening situation in any demographic; it should be noted that individuals with PWS are particularly at risk due to the comorbidities of the condition (obesity) [3]. This case report looks at an interesting case of an individual with PWS who presented with characteristic findings of an acute saddle PE and was treated successfully.

## Case presentation

**Figure 1:**
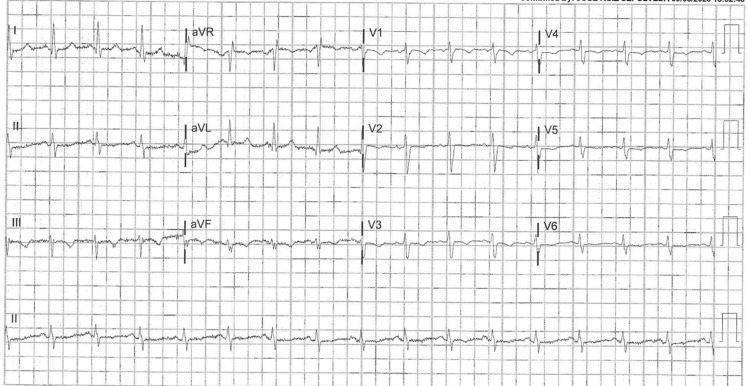
EKG: normal sinus rhythm, rate of 96

The patient in this case report was a 41-year-old male with a past medical history of PWS and morbid obesity who presented to the emergency department via emergency medical services (EMS) with a chief complaint of shortness of breath and general malaise. The patient also had a prior diagnosis of adrenal insufficiency and took chronic steroid therapy as his treatment. The patient’s parents were his primary caregivers, and he expressed that he had had these symptoms for over two weeks, but his shortness of breath had exacerbated over the two days before his arrival at the ED. The patient was brought into the ED via EMS, which reported that his oxygen saturation was stable at 92%. Upon physical examination, vitals included a blood pressure of 144/83, a temperature of 36.8°C, a heart rate of 99, and a respiration rate of 18/min. Both parents discussed that his basic daily movements of just “walking around” resulted in him having diaphoresis, lightheadedness, and fainting episodes. The mother of the patient stated that the patient did participate in exercise and made efforts to live a healthy lifestyle but had not recently due to his worsening condition. Upon further questioning, the patient denied alcohol or tobacco use, nausea, vomiting, hemoptysis, calf pain, or abdominal tenderness. The patient endorsed a dry cough, shortness of breath, and profuse diaphoresis; he had no gross visual unilateral swelling in his lower extremities. A triage EKG showed benign findings, with no sinus tachycardia, ST elevation, or right bundle branch block (Figure 1). He was started on a nasal cannula to support his oxygenation and dyspnea with 1 L of oxygen. Upon further evaluation of said symptoms, a lead diagnosis of PE was made. Utilizing the PERC-Wells criteria, the patient was not able to be “PERC’d” out, and a preliminary order of tests including a chest X-ray and corresponding blood work of the complete blood count, comprehensive metabolic panel, d-dimer, n-terminal proBNP (BNP), and troponin cardiac markers were quickly ordered (Table 1).

**Table 1:** Lab values associated with the patient ALT, alanine transaminase; AST, aspartate aminotransferase; BUN, blood urea nitrogen; CBC, complete blood count; CMP, comprehensive metabolic panel; MCH, mean corpuscular hemoglobin; MCHC, mean corpuscular hemoglobin concentration; MCV, mean corpuscular volume; MPV, mean platelet volume; RBC, red blood cell; RDW, red cell distribution width; WBC, white blood cell

CBC	Reference ranges	Admission values
WBC	4.40-10.50 10*3/uL	10.54
RBC	4.00-5.5.65 10*6/uL	4.28
Hemoglobin	12.6-16.7 g/dL	13.5
Hematocrit	36.9-48.5%	40.7
MCV	82.4-99.3 fL	95/1
MCH	27.5-34.1 pg	31.5
MCHC	31.7-36.1 g/dL	33.2
RDW	11.4-14.9%	12.50%
Platelet count	139-361 10*3/uL	219
MPV	9.7-12.5 fL	11.1 (elevated)
Neutrophils %	20.5-45%	73.9 (elevated)
Lymphocytes %	1-15%	6.8
Monocytes %	0-5%	1.2
CMP		
Sodium	135-145 mmol/L	130 (decreased)
Potassium	3.5-5 mmol/L	4.5
Chloride	98-110 mmol/L	95 (decreased)
Carbon dioxide	24-31 mmol/L	25
Anion gap	5-15 mmol/L	10
BUN	5-25 mg/dL	9
Creatinine	0.6-1.20	0.8
Glucose	70-100 mg/dL	131 (elevated)
Calcium	8.5-10.5	9
AST	4-46 U/L	34
ALT	4-51 U/L	33
Alkaline phosphatase	40-129 U/L	91
Protein, total	6.5-8.0 g/dL	6.7
Albumin	3.20-5.50 g/dL	3.9
Bilirubin, total	0.10-1.5 mg/dL	0.4
Biomarkers		
N-terminal proBNP	0-125 pg/mL	3,026 (elevated)
Troponin T, high sensitivity	≤22 ng/L	24, 24, 23 (elevated)
D-dimer	0.27-0.49 ug/mL FEU	14.93 (elevated)

**Figure 2:**
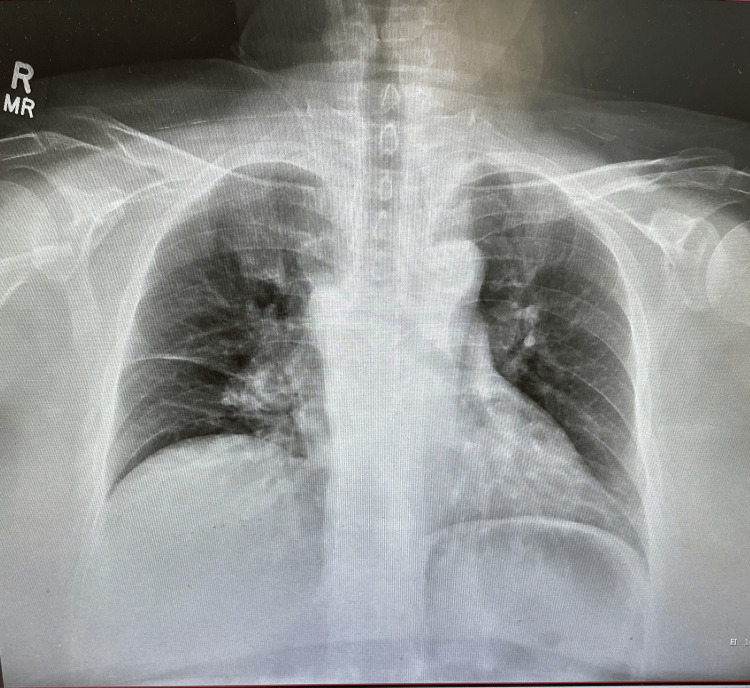
Chest X-ray of the patient showing prominence in the hilar region, an elevated right dome, and atelectasis in the right mid-lung

**Figure 3:**
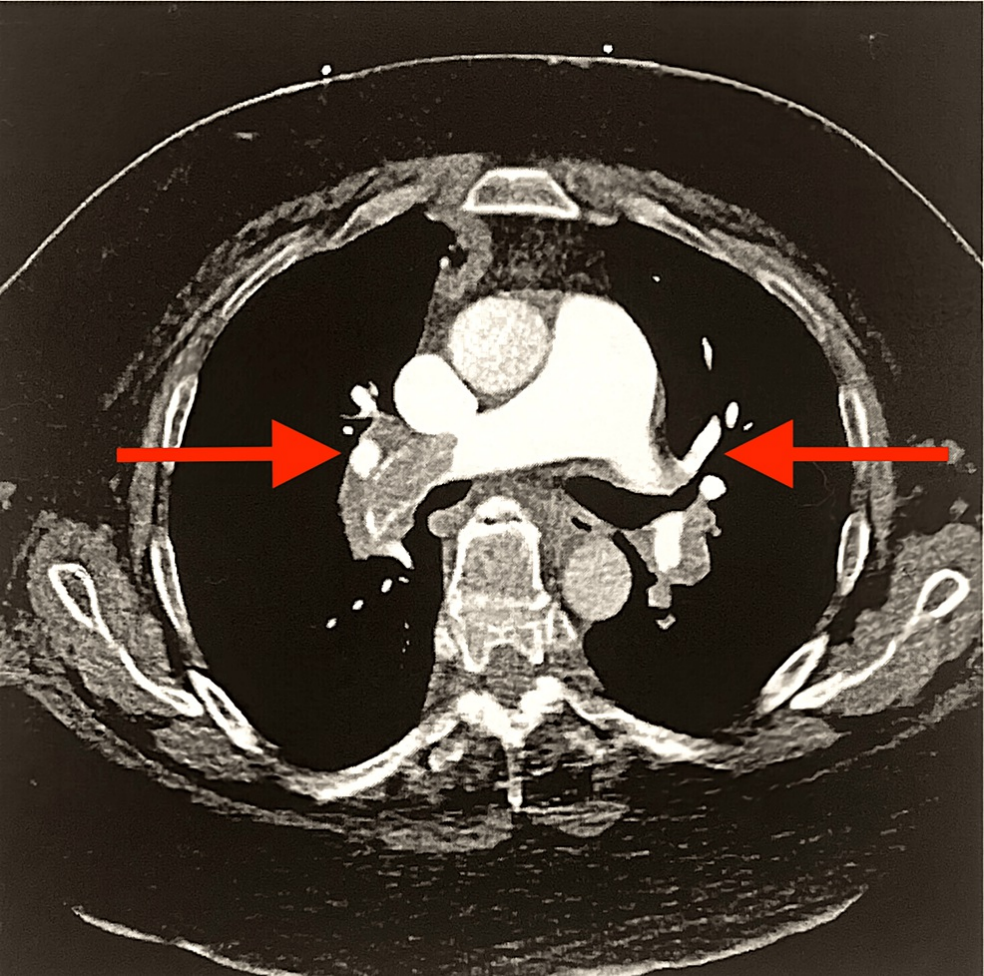
CT pulmonary angiogram showing PE PE, pulmonary embolism

**Figure 4:**
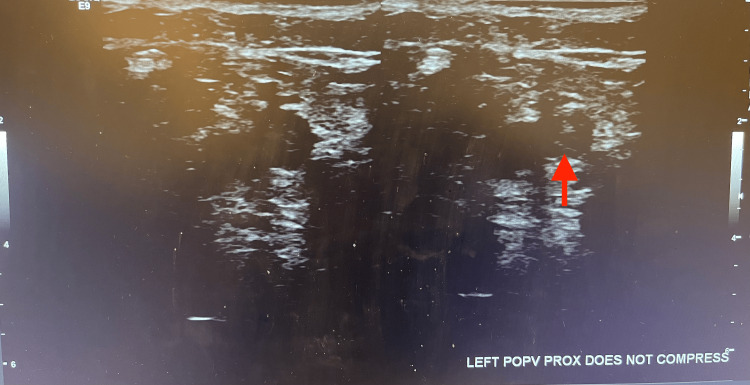
Ultrasound showing noncompressible left proximal popliteal vein DVT DVT, deep vein thrombosis

A chest X-ray was performed and showed a finding of prominence in the hilar region, an elevated right dome, and atelectasis in the right mid-lung (Figure 2). The D-dimer markers were significantly elevated at 14.93 uG/mL, and BNP was also at 3,026 pg/mL. Upon collection and evaluation of these lab markers, a CT pulmonary angiogram and a lower extremity ultrasound were urgently ordered. The attending radiologist of the CT imaging marked critical findings and remarked that there were extensive bilateral pulmonary emboli in the distal main pulmonary arteries (Figure 3); furthermore, the pulmonary artery was dilated at 3.9 cm, and the right ventricle to left ventricle ratio was 1.5 (greater than 1 indicates right heart strain). A lower extremity ultrasound also found a deep vein thrombosis (DVT) in the proximal left popliteal vein (Figure 4). Due to these imaging findings, the patient was immediately started on a heparin drip (25,000 units), per PE hospital protocol. Due to the emergent situation of the patient’s findings, interventional radiology (IR) was consulted, and the patient was discussed. The attending IR physician agreed to take the patient based on the CT findings that showed the right heart strain and pulmonary artery dilation. The patient was transferred to IR for a catheter-directed thrombolysis (EKOS) procedure in which tissue plasminogen activator and embolectomy were used to remove the emboli. The patient tolerated the procedure well and was sent to the intensive care unit for observation. A few days later, the patient was discharged after a successful recovery. The patient was not followed after discharge.

## Discussion

Pulmonary emboli, when significantly large, as in the case of this patient, can oftentimes be fatal if misdiagnosed or if treatment is delayed. It is essential to understand the dynamics of the problem and why there seems to be a high rate of PEs in this demographic. It should be noted that this patient’s age is beyond the life expectancy of individuals with PWS [2]. Patients affected by PWS are characteristically obese, and numerous metabolic pathologies such as hypertension, diabetes mellitus, and hypercholesterolemia are closely associated [4]. It can be concluded that obesity is the most significant contributor to the morbidity of patients with PWS, with cardiovascular disease having a close associational component [5]. Obesity is linked to venous thrombi, and as a result, PE is a common finding in this demographic [4]. Anecdotally, the patient’s parents and caregivers expressed that the patient exercises continuously and consistently and was treated and cared for by a significant state-affiliated academic hospital; however, upon further review of the patient’s medicine records, no findings of growth hormone were made. This is significant as growth hormone is a treatment that is often used in the PWS population, and growth hormone is also linked to venous thrombi [5]. Literature has found that even with lifestyle modifications, patients with PWS are still affected by morbid obesity [6], and often drastic measures such as bariatric surgery are used to help control the driving force of obesity, which is unsuppressed appetite [7]. Research is currently limited in the scope of the correlation of why death via PE is prominent in individuals with PWS; more research regarding the correlation should be studied as it is one of the leading causes of death in patients with PWS [4].

Chest X-rays are not usually used as a screening pattern for PEs due to their low sensitivity for PE. However, they are routinely used to rule out other processes such as pneumonia, tension pneumothorax, or even tuberculosis. The attending physician made a clinical judgment due to the knowledge that atelectasis is a possible finding with PE [8]. Another interesting finding was the presentation of the individual; the patient presented with no apparent unilateral leg swelling, which is often indicative of DVT [9], although ultrasound testing later confirmed DVT. Sinus tachycardia, S1Q3T3, and right bundle branch block [10], described as hallmark PE findings, were not noted in this patient, even though the CTA showed pulmonary artery hypertension or dilation.

Manzardo et al. concluded that DVTs are prominent in the PWS population, and the same comorbidities (obesity) that affect the general population are also associated with PWS patients [11]. It can be deduced that the patients are more likely to be exposed to PE due to the obesity associated with the syndrome. Matesevac et al. have also conducted a study that has strengthened the point that PEs and DVTs are more likely to be found in the PWS patient population than in the general population [12]. Regardless of this information, much more evidence is needed to understand why pulmonary embolus is associated with this demographic; it can be speculated that obesity and medications (hormone replacement) may be contributing factors to the incidence of PE in the PWS population.

## Conclusions

This case report highlights the importance of clinical manifestations of PE and how clinical judgment should be used when assessing patients for possible PE, even when characteristic findings such as chest pain, sinus tachycardia, shortness of breath, and unilateral leg swelling are absent. PE has many presentations, some more common than others, and all clinicians are urged to be aware of these patterns, as they can mask themselves very well. However, more importantly, given the rarity of PWS and the characteristics of the population, this article’s authors believe more case reports on PWS and its associations with PE may contribute great value to medical knowledge. The authors believe that X-rays, although not used as first-line tests for clinical suspicion of PE, should still be used in clinical decision-making. However, a good history and physician exam are much more significant, and emphasis should be placed on collecting a good history; this is often difficult given the demanding time constraints of the emergency departments nationwide. Although it can be assumed with clinical reasoning that obesity is the strongest culprit, more evidence is needed in this demographic to make a strong claim. The authors of this report urge clinicians to continue to be judicious when dealing with patients who come in for dyspnea and to remain vigilant when classic hallmark findings are absent but clinical gestalt “feels” otherwise.
